# Electrical and Electrochemical Behavior of Carbon Paste Electrodes Modified with Ionic Liquids Based in *N*-Octylpyridinium Bis(Trifluoromethylsulfonyl)Imide. A Theoretical and Experimental Study

**DOI:** 10.3390/molecules24183382

**Published:** 2019-09-17

**Authors:** Carla Báez, Freddy Navarro, Francesca Fuenzalida, María J. Aguirre, M. Carmen Arévalo, María Afonso, Camilo García, Galo Ramírez, J. Antonio Palenzuela

**Affiliations:** 1 Departamento de Química de Los Materiales, Universidad de Santiago de Chile, Av. B. O’Higgins 3363, Estación Central, Santiago 9170022, Chile; carla.baez@uach.cl (C.B.); freddy.navarro@usach.cl (F.N.); 2 NM MultiMat, Instituto de Ciencias Físicas y Matemáticas, Facultad de Ciencias, Universidad Austral de Chile, Campus Isla Teja s/n, Valdivia 5110566, Chile; 3 Instituto de Ciencias Naturales, Universidad de Las Américas, Av. 5 de Abril 0620, Maipú, Santiago 9251454, Chile; ffuenzalida@udla.cl; 4 Departamento de Química Física, Instituto Universitario de Materiales y Nanotecnología, Universidad de La Laguna, Avda. Astrofísico F. Sánchez s/n, 38071 La Laguna, Spain; carevalo@ull.edu.es; 5 Departamento de Química Orgánica, Instituto Universitario de Bio-orgánica Antonio González, Universidad de La Laguna, 38206 La Laguna, Spain; mmafonso@ull.edu.es; 6 Departamento de Ciencias Biológicas y Químicas, Núcleo de Bioproductos y Materiales Avanzados, Universidad Católica de Temuco, Avenida Rudecindo Ortega 02950, Temuco 4781312, Chile; cgarcia@uct.cl; 7 Departamento de Química Inorgánica, Facultad de Química y de Farmacia, Pontificia Universidad Católica de Chile, Av. Vicuña Mackenna 4860, Santiago 7820436, Chile; gramirezj@uc.cl

**Keywords:** ionic-liquid carbon paste electrodes, *N*-octyl pyridinium bis(trifluoromethylsulfonyl)imide, intramolecular forces of ionic liquid

## Abstract

In this work, we studied carbon paste electrodes (CPEs) with two kinds of binders: mineral oil or ionic liquids (IL) derived from N-substituted octyl pyridinium bis(trifluoromethylsulfonyl)imide with the substituents H-, CH_3_-, CN- and CF_3_-. The work aims to study this series of IL and determine a possible effect of the substituent of the cation in the behavior of the IL as a binder of graphite for obtaining IL-CPEs. The electrochemical response and the electrical behavior were measured by cyclic voltammetry and electrochemical impedance spectroscopy, respectively. Surprisingly, the substituent does not affect the cyclic voltammetry response because in all the cases, high resistance and high capacitive currents were obtained. The best response in terms of conductivity is obtained by CPE. In the case of impedance measurements, the substituent does not cause differences, and in all the cases, the IL-CPEs show nearly the same responses. CPE shows lower capacitance and higher resistance for diffusion compared to the IL-CPEs due to his lower porosity. The high resistance showed by the IL-CPEs by cyclic voltammetry can be attributed to poorly intermolecular forces among graphite, water, electrolyte, and ILs as demonstrated by theoretical calculations.

## 1. Introduction

In recent years, ionic liquids (ILs) have attracted considerable interest in the scientific community, especially in terms of sustainable or green chemistry. The modulation of their physical and chemical properties makes them versatile compounds in different areas of chemistry. The possibility of generating a great variety of combinations between cations and anions allows ionic liquids to have diverse applications, of which many are significant in the industry [1]. They have chemical stability, low vapor pressure, low melting temperature, thermal stability, and electrochemical stability since they are stable over a wide range of potentials.

Within the industrial applications where ionic liquids have presented greater use, the synthesis of organic compounds appears [2] in which no organic solvents are used. They have also been applied as catalysts for obtaining organometallic compounds [3], as separators of azeotropic mixtures, as additives in paints, as lubricants, as supported ionic liquid membranes needing a minimum amount of solvent, and as dispersants and surfactants [4].

On the other hand, because RTILs (ionic liquids which are liquids at room temperature) have intrinsic ionic conductivity at room temperature and a wide electrochemical window [5], they have also been used as electrolytes in the manufacture of polymer-based electrochemical devices and the electrochemical synthesis of conductive polymers. Among the most studied ILs in electrochemical reactions are those derived from the imidazolium cation such as -butyl -methylimidazolium hexafluorophosphate that has mainly been used as an electrolytic solution and in the modification of glassy carbon, platinum, and carbon paste electrodes (CPEs) [6]. Additionally, the ILs derived from the pyridinium cation such as *N*-octylpyridinium hexafluorophosphate, which has mainly been used in the obtaining of modified CPEs [7,8].

Within the various electrochemical applications, ILs have been studied in different types of energy storage devices such as batteries and supercapacitors, due to their low vapor pressure in Li-S type batteries, Li-air batteries and supercapacitors, and in solid-state batteries, ILs can be used to improve the conductivity of the solid electrolytes [9].

The possible combination of cations and anions generate many ILs. Among the possible combinations, fluorinated anions with alkylimidazolium-type cations [10] have resulted in ILs that show relatively wide potential windows, low viscosity values, and high conductivity values. These favorable characteristics allow their use in electrochemical studies [11].

Another of the physical properties that can be modulated when using different anions and cations is the melting point of ionic liquids. In general, an increase in the size of the anion or cation decreases the melting point values. That is, the greater the delocalization of the charge, the lower the melting point of the ionic liquid [12,13].

The miscibility of the IL with water depends strongly on the anion and to a lesser extent on the cation [14]. The most hydrophobic ILs are obtained by using anions such as bis(trifluoromethylsulfonyl) imide (NTf_2_-) and hexafluorophosphate (PF_6_-), while the use of tetrafluoroborate anion (BF_4_-) generates more hydrophilic ILs. Additionally, bulky cations substituted with long alkyl chains have limited water solubility [15]. The amount of water absorbed by ILs depends strongly on their hydrophobicity, that is, the greater their hydrophobicity, the lower their absorption capacity [16,17,18].

In theory, it is possible to design an IL to obtain a specific set of physicochemical properties such as melting point, viscosity, density, water-solubility, catalytic activity, and selectivity [19,20]. However, the design is complex because the physical-chemical properties of the ILs vary in a non-linear way, which makes the study of the effects produced on the properties of the ILs due to changes in the cation and the anion still in its beginning, despite the relevance of this topic.

In a previous work [21], we studied the electrical and electrochemical behavior of a graphite powder electrode bonded with a series of ILs derived from 4-substituted-*N*-octylpyridinium hexafluorophosphate. Carbon paste electrodes are very low cost, renewable surface electrodes, easy to modify, and widely used in electroanalytical and electrocatalytic applications [22,23,24].

In general, carbon paste electrodes are composed of graphite powder, conductor, and a binder such as silicone or mineral oil, which does not conduct the current. To increase the conductive characteristics of these electrodes, the use of ionic liquids as binders is a good alternative. If the electrode is used in an aqueous medium, it is convenient that the ionic liquid has rather hydrophobic characteristics so that it is stable in the medium, so a bulky anion is necessary, such as hexafluorophosphate and a cation that has some alkyl chain, such as *N*-octylpyridinium [21]. In this way, highly conductive and renewable surfaces are obtained. In that sense, the use of *N*-octylpyridinium bis (trifluoromethylsulfonyl) imide arises as a viable alternative for the modification of carbon paste electrodes.

**Figure 1:**
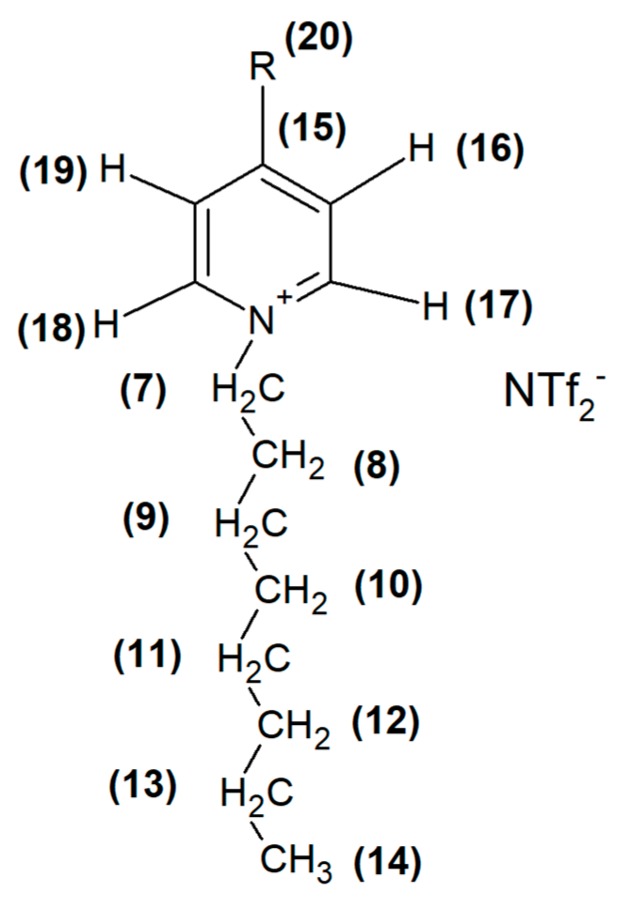
Schematic representation of the ionic liquids (ILs) where R is H-, CH_3_-, CN-, or CF_3_-. Proton-label is shown.

In our previous study, we found significant differences (in an electrochemical and electrical sense) when electron-donating or electron-withdrawing substituents substituted the cation of the IL. In this work, we study the same cations but varying the anion, in this case, NTf_2_- (see Figure 1). The work aims to understand the chemical nature of the interactions of cations and anion, its behavior as a binder for CPE, to predict if an ionic liquid will be able to be used for electrochemical purposes when varying the interactions between cation and anion.

## 2. Results

### 2.1. Characterization of ILs

The synthesis of all the IL were realized as described in Materials and Methods. Ionic liquids were obtained pure and characterized by ^1^H-NMR, ^13^C-NMR, and FT-IR.

Figure 1 shows the structure of the ILs obtained.

Table 1 and Table 2 show the signals corresponding to the ^1^H-NMR and ^13^C-NMR spectra of all the ILs, respectively. 

Table 3 shows signals corresponding to the FT-IR spectra for all the IL. Absorption bands between 2900 and 3200 cm^−1^ correspond to νCsp2-H of the pyridinium ring and νCsp3-H of the alkyl chain. At lower values of frequency, 1430 and 1650 cm^−1^ appear the absorption bands νC = C y νC = N corresponding to the pyridinic ring [21]. At lower frequencies appear the vibrations corresponding to the anion [25].

Results obtained from melting points and Table 1, Table 2 and Table 3 confirm that pure ILs were obtained.

### 2.2. Electrochemical Behavior

For a perfect conducting system, when a potential is applied and there are not any redox species in the solution, the response of the system must be zero current. Conversely, if the system presents resistance, then Ohm’s law predicts a behavior where the current varies according to the applied potential, depending on its resistance value. In this sense, it is very noticeable the very resistive response of the CPE modified with any of these ILs compared to the response of a conventional CPE at pH 3.0 using Britton–Robinson buffer (BR). It is not easy to explain the resistive response obtained because ILs are more conductive than the mineral oil [5,16,26].

**Figure 2:**
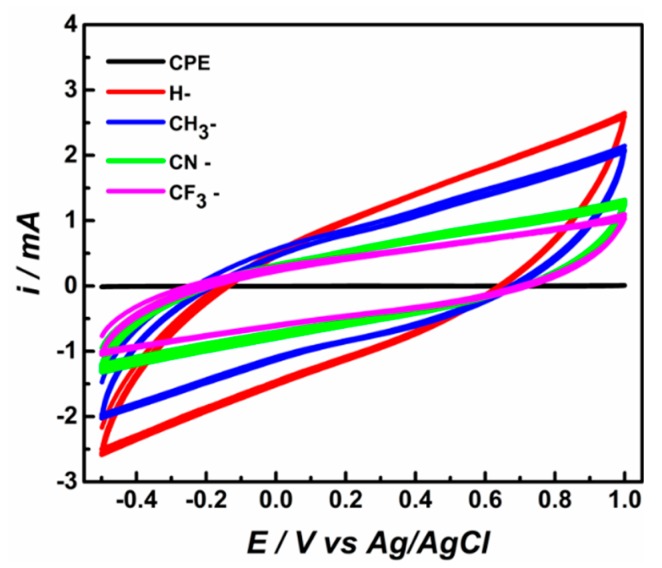
Voltammetric response of carbon paste electrodes (CPE) and modified electrodes. Experiments realized in an Ar-bubbled solution of Britton–Robinson buffer (BR) at pH 3. Scan rate: 100 mVs^−1^.

The voltammetric response indicates that the entire electrode is not capable of conducting the electric charge showing a response that corresponds to a resistance (see Figure 2). In this figure, CPE does not present a resistive response because the slope of the graph I vs. E is zero.

For the electrodes modified with ILs, the response in current is wide and presents a rectangular box-like shape. In fact, for a defined value of potential, 0.2 V as an example, the current ranges from −1.5 to 1 mA for the H-system. This behavior is due to the rearrangement of the double-layer of the solution in contact with the surface that generates this response of capacitive current (that does not correspond to a faradaic process) [27]. A wider response corresponds to a high rearrangement. It is clear from Figure 2 that the response of each electrode presents high capacitive currents compared to that of the conventional CPE (that appears as a line for scale reasons). The capacitive response is probably due to a very porous surface compared to that corresponding of CPE. In Figure S1 the SEM images of CPE and electrodes modified with ILs are quite similar, despite the compound used as a binder, but electroactive areas from ILs modified electrodes are larger than CPE [7,8]. The electroactive area was measured according to the Randles–Sevcik equation (Equation (1)) obtained for the outer-sphere redox couple (K_4_ [Fe (CN)_6_]/K_3_ [Fe (CN)_6_]) [2,21] and they are shown in Table 4.

i_p_ = (2.69 ∗ 10^5^) n^3/2^ D^1/2^ ν ^1/2^ AC, (1)

where D is the diffusional coefficient of Fe (II) (7.6·10^−6^ cm^2^/s) [25], n is the number of transferred electrons (n = 1), C is the concentration of the active species Fe (II)/Fe (III) (2 mM), A is the electroactive area, and ν is the scan rate. According to that couple, the electroactive areas were measured, and they are shown in Table 4. Additionally, the roughness factor R can be measured as the ratio between the electroactive and the geometrical area. For each case, the geometrical area was 0.031 cm^2^.

The roughness factor for all the electrodes modified with ILs are close to 2.5, indicating a porous surface. CPE has an electroactive area practically equal to the geometrical area that corresponds to a smooth surface. It is important to mention that the voltammetric response remains stable when the electrode is immersed after being exposed to air for 24 h, indicating that the surface and the roughness maintain its values. From these results it is possible to infer that in the case of the electrodes modified with ILs, pores are generated and when the electrode is immersed in the solution, water enters through the pores of the surface due to the more hydrophilic nature of the ILs, preventing an adequate interaction between a particle of graphite and another. Figure S2 compares the behavior of a water drop on the surface of the H-modified electrode and CPE, showing clearly that the IL-modified electrode has a more hydrophilic nature compared to the CPE. The other IL-modified electrodes show a very similar response to that of H-modified electrode and they are not shown. For those reasons, the voltammetric response of the electrodes modified with the ILs have a high capacitive current response and a resistive character. There are slight differences (in both capacitive and resistive response) among the different electrodes due to the differences between the hydrophilicity of the ILs and the nature of the interactions between cation and anion. 

### 2.3. Electrical Behavior 

**Figure 3:**
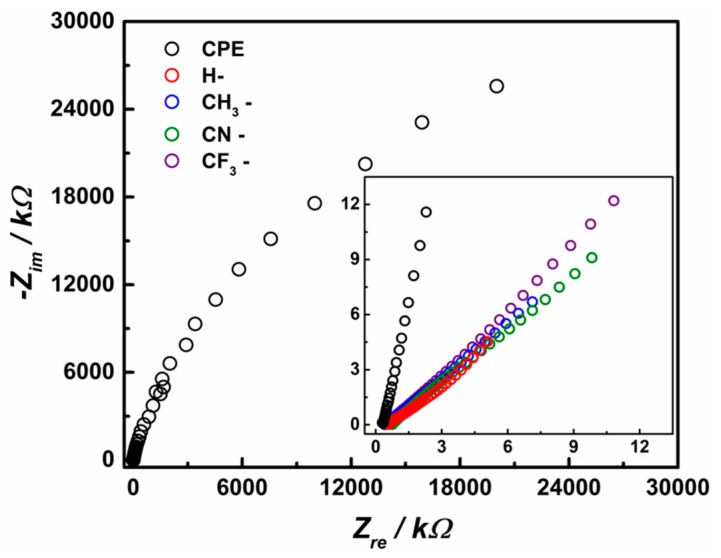
Nyquist plot for CPE and ILs modified electrodes. Frequency range: 100,000–0.01 Hz. Sinusoidal potential pulse = 5 mV. All measurements at open circuit potentials. Britton–Robinson buffer pH = 3.00.

It is possible to measure the whole electrical properties of the electrode/electrolyte interface through the electrochemical impedance spectroscopy, EIS. With this technique, the whole system can be represented by an electric circuit that fits the observed response. The response is generated by small perturbations of an alternating potential around a fixed value when the frequency of the perturbation changes. These changes can be associated with different circuitry elements that could vary its impedance with the frequency of the perturbation. Nyquist graphs (imaginary versus real impedance) show in the real axis, the elements of the circuit that do not depend on the frequency. In the first place appears the resistance of the solution at high frequencies and then, the resistance of the system to a charge transfer process. Then, more parallel to the imaginary axis, the elements of the circuit that depends on the frequency appear (diffusion and the capacitor behavior). Figure 3 corresponds to the response of all the systems. For scale reasons only CPE appears. The inset of Figure 3 shows the amplified zone of the beginning of Figure 3, exhibiting for all the ILs the resistance to ion migration at higher frequencies and then all the spectra increase in both axes, suggesting a diffusional process very similar for all the electrodes modified with the ILs despite the nature of the substituent. In the case of CPE (Figure 3, inset), the spectrum increases in a first instance parallel to the imaginary axis and then impedance at both axes increases. This behavior could be related to resistance to electron transfer, but in this case, there is no redox probe in the solution, then no electron transfer can occur. It is not easy to understand this behavior with only these results. For that reason, Bode graphs were also analyzed. 

**Figure 4:**
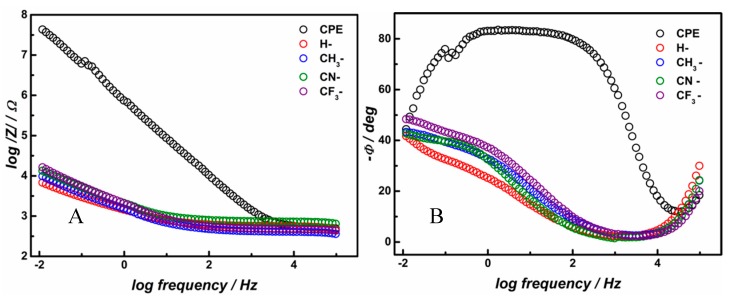
(**A**,**B**) Bode plot for CPE and ILs modified electrodes. Frequency range: 100,000–0.01 Hz. Sinusoidal potential pulse = 5 mV. All measurements at open circuit potentials. Britton–Robinson buffer pH = 3.00.

Analyzing Bode plots (Figure 4A), it can be observed that the capacitance is lower for CPE compared to all ILs because the module of impedance is defined inversely proportional to the capacitance [28]. This behavior agrees with the high capacitive-current response that the IL-modified electrodes present in the cyclic voltammetry. In the Bode graphs, the similarity among the IL-electrodes despite the substituent of the cations is evident. All of them present similar responses except for the CPE electrode, as it was mentioned.

**Scheme 1:**
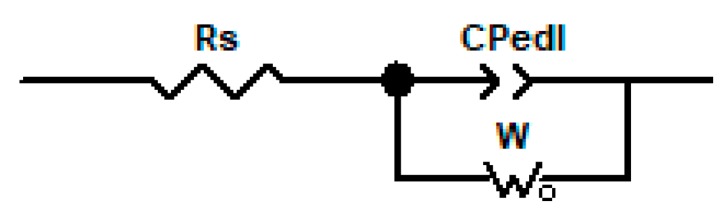
The equivalent circuit proposed. Rs = resistance of solution, CPedl = constant phase element, W = Warburg impedance.

On the other hand, as can be seen in Figure 4B, at low frequencies all the spectra finish in an −45° phase angle, then, this process could be associated with the charging of the double layer in parallel to a diffusional process through the surface because an ideal capacitor reaches a phase angle of −90° at these low frequencies [28]. Resistance to electron transfer is not observed in all frequency range studied as expected. In addition, Figure 4B shows that CPE increases its phase angle when frequency varies, what is associated with a resistance parallel to a capacitor due to the behavior of the double layer in the interface. Additionally, all ILs systems show angles lower than −10° at frequencies higher than 1000 Hz that correspond to a resistance behavior and at lower frequencies they reach angles close to −45°. The proposed equivalent circuit is described in Scheme 1, and the results are summarized in Table 4. In this circuitry, R_s_ is associated with the solution resistance, CPe is a constant phase element that represents the electric double layer that is modeled especially in place of an ideal capacitor to represent non-homogeneity of electrodic surfaces. The impedance of the CPe (Z_CPe_) is defined by Equation (2).

Z_CPe_ = 1/[T(i·ω)^P^], (2)

where ω is the frequency and P and T are parameters that account for the electrical behavior. When P is 1, the T value is considered the capacitance value of the electrical double layer. This constant phase element is connected in parallel to an open circuit Warburg element, like a Randles circuit [28]. This element represents the diffusion process through the CPE. The presence of this circuitry element was considered based on the behavior of the Nyquist plots where all systems studied are not parallel to the imaginary axes as an ideal capacitor. In addition, in the Bode plots, all of them finished at an angle phase close −45°, hence the impedance value at low frequencies can be associated with the charging of the double layer in parallel to diffusional processes through the surface. This Warburg element is defined by Equation (3) [28].

Z = W_o_-R·ctnh ([I·T·ω]^P^)/(I·T·ω)^P^, (3)

where W_o_-R is the resistance of Warburg and was considered as free to the fitting, P was considered fixed at 0.5 for a diffusional process, and T is fixed because it is related to a length of the diffusional layer and coefficient diffusion value (L^2^/D) and I is the current. 

Table 5 shows the values of the parameters obtained with the equivalent circuit shown in Scheme 1. It can be observed that capacitance (measured by T) is low for CPE compared to ILs. The value for Warburg resistance of the CPE is one order of magnitude higher compared to all ILs. This result agrees with the low porosity of the CPE compared to the modified ones. As it was mentioned before, there is a slight variation in capacitance and Warburg resistance with the change of substituent in ILs. The main difference is observed in capacitance and resistance in the Warburg element when comparing the CPE and all ILs. Capacitance is related to the electrical double layer, and resistance in the Warburg element in this work could be associated with the ability of the system to diffuse through the surface of the electrode, under acidic conditions. 

In order to understand the lower ionic characteristics of the NTF2-ILs and the low influence of the substituent of the cation in this behavior, the theoretical calculation was made.

### 2.4. Electronic Structure Calculations

**Figure 5:**
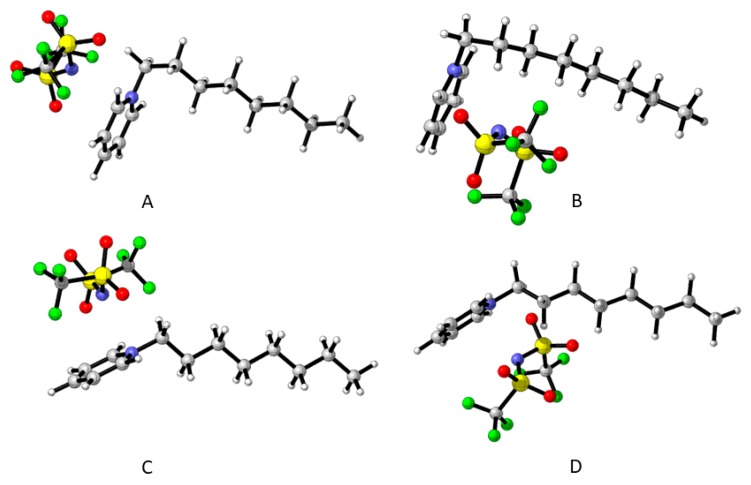
The four spatial arrangements studied. (**A**) The structure where cation with the alkyl chain is perpendicular to the aromatic ring and the anion outside of the cavity. (**B**) The same structure from A, but the anion is inside the cavity. (**C**) The cation is almost lineal, and the anion is outside of the cavity. (**D**) The cation is the same structure that C, but the anion is inside of the cavity. Only the unsubstituted pyridinium derivatives are shown for clarity.

In order to explain the behavior of the ionic liquids studied, we started by analyzing their structure using DFT calculations. Previous work on similar ionic liquids [21] has shown that the anion and the cation can adopt different relative dispositions with little energy difference that can exist in equilibrium. A small number of such structures are then considered to extract information about the properties of the ionic liquid in the gas phase. In this work after considering several arrangements calculated at the semiempirical level, we decided to use the two more important conformations of the 4-substituted-*N*-octylpyridinium cation and to place the anion either in the outside or in the inside of the cavity formed. The anion studied bis(trifluoromethylsulfonyl)imide also has some complexity since it has some conformational mobility and thus can interact in different ways with the cation. The lower energy structures found for each relative disposition of anion and cation were used for this study. This resulted in the four general structures depicted in Figure 5.

Table 6 shows the relative energies of all the spatial arrangements for the four ionic liquids studied. In all cases, two of the arrangements, B and D are more stable than the others, although by a small amount (from 1.23 to 3.02 kcal/mol). This can be explained by the possibility that exists in structures B and D of the anion forming multiple non-bonding interactions with the alkyl chain of the cation, a possibility that does not exist for A and C.

This is, of course, a very partial vision of the behavior of the bulk ionic liquid, but its utility in that it gives an idea of the different equilibrium structures that may exist and the interaction between the anion and the cation.

To support this explanation, a study of the Non-Covalent Interactions (NCI) of the four ionic liquids was undertaken. The NCI were studied using both the Quantum Theory of Atom in Molecules (QTAIM) [29] and the Reduced Density Gradient (RDG) [30].

#### 2.4.1. QTAIM Analysis

QTAIM studies show several bond critical points (BCP) of non-covalent nature, indicated by a small electron density and a small-positive Laplacian of the electron density. The number of BCP of this type between anion and cation is slightly different in each arrangement, ranging from 7 in C to 11 in B for the unsubstituted pyridinium. The main difference being the interaction of the anion with the alkyl chain in B and D as opposite to A and C, where the non-bonding interactions are between the anion and the pyridinium ring exclusively.

**Figure 6:**
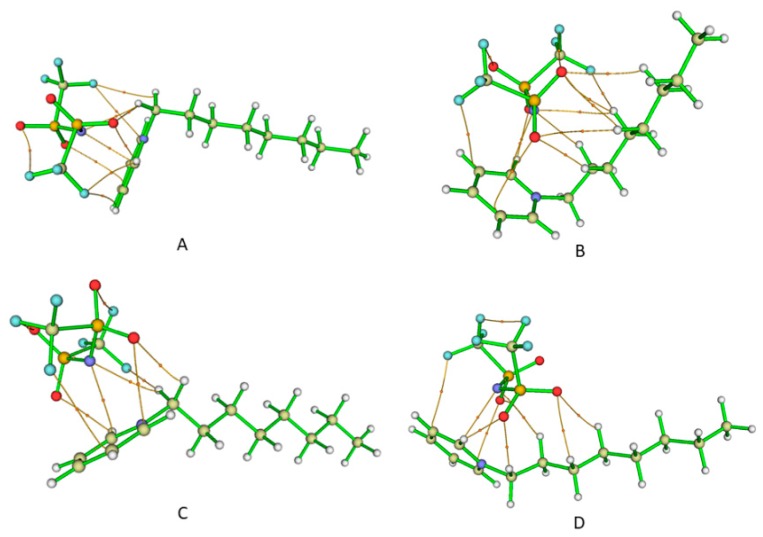
Non-covalent bond critical points (BCP) and critical bond paths for the ionic liquid in the four arrangements studied. (**A**) The structure where cation with the alkyl chain is perpendicular to the aromatic ring and the anion outside of the cavity. (**B**) The same structure from A, but the anion is inside the cavity. (**C**) The cation is almost lineal, and the anion is outside of the cavity. (**D**) The cation is the same structure that C, but the anion is inside of the cavity. Only the unsubstituted pyridinium derivatives are shown for clarity.

Figure 6 shows the four arrangements studied for the unsubstituted cation with the bond critical paths found.

**Figure 7:**
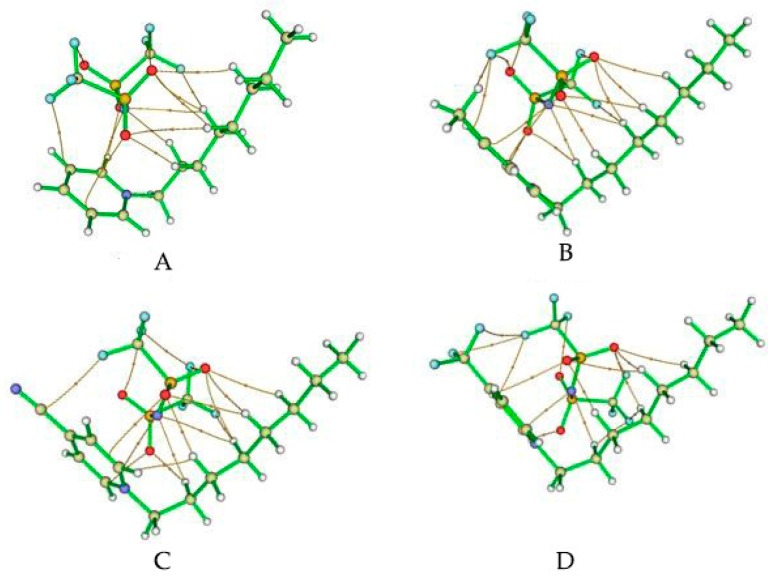
Non-covalent critical bond points and critical bond paths for the four ionic liquids studied in the B-type arrangements. (**A**) Unmodified IL; (**B**) CH_3_- modified IL; (**C**) CN- modified IL; (**D**) CF_3_- modified IL.

The same result was obtained with the other studied ionic liquids. Figure 7 shows a comparison among the four ionic liquids in the same disposition corresponding to the B arrangement.

It can be observed that in the ionic liquids with substituted pyridinium rings, extra interaction between the fluorine atoms of the anion and the substituent at the position 4 of the ring exists, helping the stabilization of those structures. The number of non-covalent BCPs formed in each ionic liquid is shown in Table 7.

The larger number of BCP is found in the B arrangement where the anion can interact with both the pyridinium ring and the alkyl chain.

#### 2.4.2. Reduced Density Gradient Analysis

**Figure 8:**
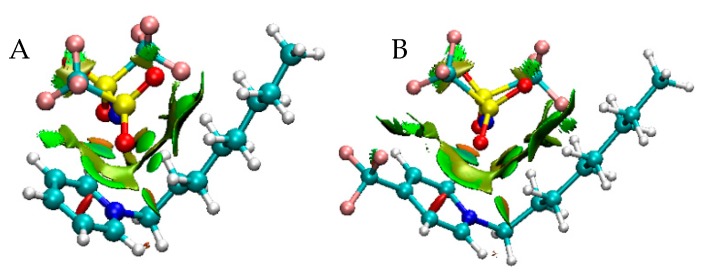
Reduced Density Gradient (RDG) plot of the ionic liquid in arrangement B. (**A**) Unmodified IL; (**B**) CF_3_- modified IL.

The difference in the number of non-covalent interactions and their nature can also be visualized using the Reduced Density Gradient (RDG) analysis. This type of analysis presents visually the region of weak interactions between two molecules based on the RDG function, which depends on the values of the electron density (ρ) and of the gradient norm of the electron density (|∇ρ|) in the form RDG = |∇ρ|/2(3π^2^)^1/3^ρ^4/3^. The isosurface of weak interaction is the area where ρ is small. |∇ρ| is 0 to small, and the value of RDG is also 0 to medium. Applying this analysis to the ionic liquids, graphs such as those shown in Figure 8 are obtained.

The color code for the interactions shown in Figure 8 is blue for H-bonds (strong interaction), green for Van der Waals interactions and red for steric repulsions. In Figure 8, using H-B and CF_3_-B as examples, most of the interactions between anion and cation are Van der Waals interactions (green). No strong H-bond is observed (blue), and a steric repulsive interaction is found between the atoms of the pyridinium ring (red).

**Figure 9:**
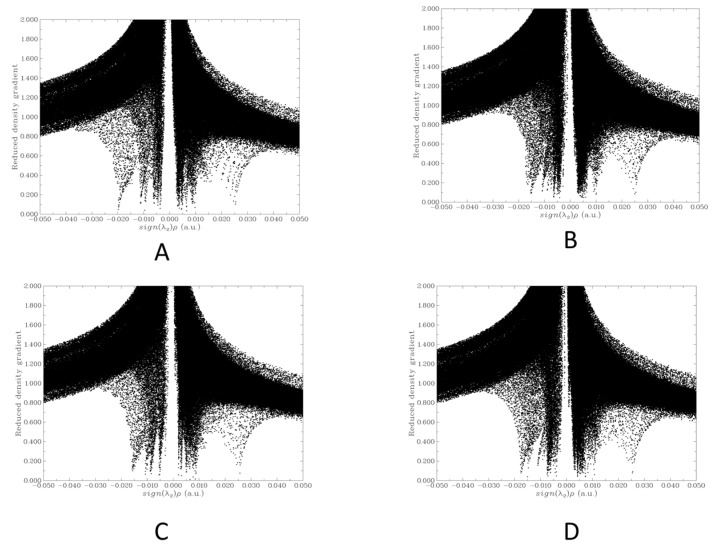
Scatter plot of the RDG analysis of the four arrangements of the unsubstituted (R = H) ionic liquids. (**A**) The structure where cation with the alkyl chain is perpendicular to the aromatic ring and the anion outside of the cavity. (**B**) The same structure from A, but the anion is inside the cavity. (**C**) The cation is almost lineal, and the anion is outside of the cavity. (**D**) The cation is the same structure that C, but the anion is inside of the cavity

In this type of analysis, the scatter plot of sign [λ_2_]ρ versus RDG where sign [λ_2_]ρ means the sign of the second largest eigenvalue of electron density Hessian matrix at a given position, gives the results shown in Figure 9.

The number and position of the spikes reaching the bottom of the plot indicate the number and nature of the non-covalent interactions in the system. The spikes to the left (negative part of the axis) indicate the existence of H-bonds or strong non-bonding interactions. Those in the center of the plot are representative of Van der Waals interactions, and those to the right (positive part of the axis) appear for steric repulsions. In the ionic liquids, there are attractive interactions, although not as strong as an H-bond. Several Van der Waals interactions can be observed, as well as a spike indicating a relatively strong steric repulsion, in good agreement to the isosurface plot shown in Figure 8.

The QTAIM and RDG analysis performed, indicates that the ionic liquids based on 4-substituted octylpyridinium cation and bis(trifluoromethylsulfonyl)imide anion presents many weak interactions, mostly of Van der Waals type due to the nature of the anion, containing several groups capable of interaction with the pyridinium ring as well as with the hydrogens of the alkyl chain. It can be expected that in the condensed phase, those interactions would change the intermolecular behavior of the ionic liquids, resulting in different properties at the macroscopic level than those shown by the same cations with other anions.

#### 2.4.3. Interaction Energy

Some macroscopic properties of the ionic liquids, such as the melting point, have been related to the interaction energy of the individual molecules in the gas phase [21], and thus the interaction energy, corrected for the Basis Set Superposition Error (BSSE) by means of the counterpoise method (CP) [31] was calculated for all the structures in this study. For each ionic liquid, the energies of the four spatial distributions of anion and cation are quite similar, and thus the average value was used for the comparison (standard deviations <3 kcal mol^−1^). Table 8 shows the computed values together with the experimental melting points. 

The values for the interaction energies shows the expected trend with the nature of the substituents on the pyridinium ring [21] for the electron-withdrawing groups (R = CN and R = CF_3_), resulting in the stabilization of the ionic pair compared to the non-substituted case (R = H) since it favors the electron transfer from the anion to the cation. The weak electron-donating group CH_3_- do not follow this trend, being slightly higher than the unsubstituted case.

The melting points of the ionic liquids with NTf_2_ as the anion are lower than those reported when PF6 is used as anion [21], and the corresponding interaction energies are also lower. The same trend for the ionic liquids with PF_6_ is observed. At least in that, the electron-withdrawing substituents resulted in higher melting points than in the unsubstituted case.

## 3. Discussion

According to the theoretical studies, the cations and anion of ILs are bonded mainly by Van der Waals interactions in addition to the electrostatic forces between anion and cation. No hydrogen bonds are observed. These strong intramolecular interactions can result in weaker intermolecular interactions with other ILs or with the environment. This can explain the macroscopic properties observed, such as lower melting points than ILs with other anions and also the higher resistivity observed in the electrodes modified with these ILs.

In fact, theoretical studies and the calculated roughness factor can explain the voltammetric and electrical behavior of the electrodes modified with the ILs. They are porous, and water can enter inside the electrode. Due to the nature of the intermolecular forces among the ILs and the surround, the interactions between the different particles of graphite are hindered. Then, they show a capacitive current, a resistive behavior, and diffusional characteristics that agree with these weak intermolecular forces.

The EIS results are in concordance with those obtained from the voltammetric response. In fact, the electrodes modified with ILs are non-ideal capacitor/resistors that allow the diffusion of electrolyte, giving a high capacitive current response. This means that the ILs that are acting as binders of the electrodes promotes the generation of holes (pores) in the electrode, where electrolyte can enter and leave the electrode. These holes are hydrophilic due to the presence of ILs, then water that enter in the electrode disfavored the contact of graphite, making the system more resistive than graphite bounded with mineral oil. This behavior is different from that presented by the same cation with hexafluorophosphate as the anion, for example, when acting as a binder of CPE electrode. In those cases, the holes are hydrophilic, but the entire electrode is conductive, because of the “more ionic” nature of the ILs that permits an electric contact among graphite particles. The term “more or less ionic” describe the possible interactions of the ionic pair with the molecules or ions from its environment. Then, in our case, the ILs with NTf_2_- as the anion has a low “ionic” behavior that is not capable of allowing the electrical conductivity along the mixture. In contrast, CPE where a graphite particle is strongly bonded to another, the electrode does not generate pores (its surface is smooth, and the electroactive area is very similar to the geometrical area) and for that reason, it does not generate high capacitive current response and shows a better conductive behavior despite the hydrophobic nature of the electrode. 

It is interesting to mention that Tokuda et al. (2004) calculate the Molar Conductivity based on ionic conductivity and ionic self-diffusion coefficients and the Nernst–Einstein equation of a series of ILs varying the anion with the same cation and the low values were obtained for NTf_2_- even lower than the case of the anion (C_2_F_5_SO_2_)N- [32].

Additionally, Yee et al. (2013), through molecular dynamics simulations, studied whether five ILs are associated or not at high dilutions. They studied C_2_mimNTf_2_; C_4_mimNTf_2_; C_6_mimNTf_2_; C_2_mimC_2_H_5_SO_4_ and C_2_mimCl. In the case of anion NTf_2_-, they exist predominantly in an associative state where the strength of the association increases with the increase of the alkyl chain of the cation. In this case, the potential of mean force (PMF) of each ion pair was calculated. They found two free-energy minima for each IL. The first appears at 3–4 Å and the second at 5–6 Å. These minima correspond to stable forms of the associated cation-anion. The depths of the relative minima and the energy barriers between them provided information about the association of ion pairs. In the case of the NTf_2_- the minima are deep, and the barrier is low then, they tend to stay associated in two arrangements, and no interactions with water are energetically favorable. Results also show that water preferably interacts with the anion. In the case of NTf_2_-, it shows a large range of depletion of water, suggesting unfavorable hydration of the anion [33].

Cammarata et al. (2001) calculate the molecular states of water in RTILs through ATR and transmission IR studies. They also demonstrate that water interacts with the anion of ILs through a bonded complex anion-HOH-anion. However, in this case, the anion NTf2- was not studied [34]. This information agrees with results observed here. We found a resistive behavior and strong interactions between cation and anion that suggest that the IL is in an associated state and only weak interactions with the surrounding water are permitted. In this case, it is expected a low ionic conductivity and also diffusion of electrolyte through the surface because water is “free” for entering or leaving because the interaction with the IL is very weak.

These results are surprising because normally ILs are described as a better binder for CPEs because they increase the area of the electrodes and enhance their conductivity [35]. According to our results and compared to our previous work [21], this description only is effective for ILs that permit intermolecular interactions. We found for ILs made with the same cations and hexafluorophosphate as anion, better responses compared to CPE (in terms of voltammetric and electrical behavior) but in that case, the interactions between cations and the anion were mainly through hydrogen bonds and electrostatics). In that case, the ILs increase the electroactive area generating pores in the surface, but the more ionic nature of the ILs (that relates with the variation of the melting points when changes the nature of the substituent) permits in the presence of water, to maintain the interactions among the graphite particles. In the present case, the “less” ionic nature of the NTf_2_- ILs relates with the low or insignificant effect of the substituent of the cation. Despite its electron-withdrawing or electron-donor nature, the surface of the electrode is practically the same. The melting points are in a narrow range, and they show practically the same electrochemical and electrical response. Further studies are necessary to conclude that the variation of the melting points with the nature of the substituents can be used as a parameter to determinate if the IL will or will not be able to be used for electrochemical purposes, due to the complexity of the interactions between cation and anion, and the effect of the molar mass of the IL in the melting point values. 

## 4. Materials and Methods 

### 4.1. Reagents and Equipment

All the reagents were analytical grade from Sigma-Aldrich (St. Louis, MO, USA) or Merck (Germany). Milli Q water was employed for all the experiments (R = 18 MΩ). Ultra-pure (99.995%) N_2_ gas (Linde, Chile) was used for bubbling the solutions before and during the electrochemical experiments. All the electrochemical measurements were done at room temperature using a one-compartment glass cell. The working electrode was a hollow Teflon tube filled with a mixture of graphite and binder in a proportion of 70:30 *m*/*m*, with a copper electrical connection. The counter electrode was a Pt wire, and the reference was Ag/AgCl (3M KCl). Ar (99.992%, Linde, Chile) was used to remove oxygen from the electrochemical measurements. All the electrochemical measurements were made in a 0.04M Britton–Robinson (BR) solution adjusted at pH 3. NMR spectra were recorded with an AVANCE 500 Bruker equipment (Bruker, Billerica, MA, USA). FT-IR were measured with an IFS 66v Bruker spectrophotometer (Bruker, Billerica, MA, USA). Melting points were measured with IA9100 Electrothermal equipment (Electrothermal, UK). SEM images were obtained with a Jeol JSM 6300 (Oxford Instruments Microanalysis Group) scanning electron microscope (Jeol USA, Peabody, MA, USA). The electrochemical and electrical measurements were done using a 604C CHI potentiostat (CH Instruments, Austin, TX, USA).

### 4.2. Synthesis of the Ionic Liquids

Pyridine (substituted or not) was mixed with bromooctane (equimolar quantities) in dichloromethane (DCM) by reflux with agitation during 48 h for pyridine and 4-methylpyridine and 96 h in the case of 4-trifluoromethylpyridine and 4-cyanopyridine. The obtained salts (with bromide as the anion) were simply mixed with equimolar quantities of LiNTf_2_ in a mixture of 1:2 v/v DCM/water solution at 25 °C with constant stirring during 2 h. The resulting mixture was separated into two phases, and the organic phase was washed with water until all the bromide disappears. Then, the solvent was eliminated, and the expected ILs were obtained. They were dried at 80 °C for 24 h [36]. In the case of the non-substituted (H-) and the CH_3_- ILs (liquids at room temperature), they were purified using a celite chromatographic column where the eluent was DCM. Additionally, for the CH_3_- substituted IL, a second column was used containing silica gel and using as eluent DCM/methanol 95:5 *v*/*v* [37]. In the case of CF_3_- and CN- substituted ILs (solids at room temperature), they were purified by recrystallization using ethyl acetate/acetonitrile 6:1 *v*/*v* [38]. Overall yields were: 48.4% for H-; 53.5% for CH_3_-; 47.0% for CN- and 50.2% for CF_3_-.

The obtained melting points of the ILs were 28 °C for CN-; 30 °C for CF_3_-. The ILs H- and CH_3_- were liquid at room temperature (25 °C). They were frozen at −18 °C and then their melting point was determined. They were −12 °C for H- and 7 °C for CH_3_-. The melting range in all cases was ±0.5 °C.

### 4.3. Electrochemical Measurements

Cyclic voltammetry was made in BR 0.04 M at pH 3.0 and room temperature, deaerated with N_2_-bubbled solution, between −0.5 and 1.0 V vs. Ag/AgCl (3 M KCl) at a scan rate of 0.1 Vs^−1^. Similar conditions were used for electroactive areas calculations, adding 2 mM K_4_[Fe (CN)_6_]/K_3_[Fe (CN)_6_] redox couple and varying scan rates from 0.005 to 0.5 Vs^−1^.

Electrochemical impedance spectroscopy was realized at open circuit potential using Britton–Robinson buffer at pH 3.0 and room temperature. A sinusoidal voltage perturbation of amplitude 5 mV was applied, scanning in the 100,000–0.01 Hz frequency range with 12 points per frequency decade. The impedance data were analyzed and fitted to electrical equivalent circuits with ZView 3.5f software (Scribner Associates Inc., Southern Pines, NC, USA).

### 4.4. Computational Methods

All calculations were carried out using the GGA functional BP86 and the def2_TZVP basis set [39] as implemented in ORCA 4.1 (Max-Planck-Gesellschaft zur Förderung der Wissenschaften e.V. (MPG), Germany) [40]. To account for weak interactions, the D3BJ dispersion correction was applied [41]. Frequency calculations were used to ascertain the nature of the stationary points calculated.

The QTAIM and RDG analysis were carried out using the Multiwfn v3.6 software (Dr. Tian Lu, China) [42]. The interaction energies were calculated subtracting the energies of the cation and anion from the energy of the ionic liquids and were corrected for basis set superposition error (BSSE) using the Boys and Bernardi procedure. Plots of RDG isosurfaces were obtained using VMD v1.9.3 software (Theoretical and Computational Biophysics Group from University of Illinois at Urbana-Champaign, Urbana-Champaign, IL, USA) [43].

## 5. Conclusions

The results show that carbon paste electrodes modified with the series of ionic liquids derived from N-substituted octyl pyridinium bis(trifluoromethylsulfonyl)imide with the substituents H-, CH_3_-, CN-, and CF_3_- shows a poorly electrochemical behavior due to the capacitive and resistive response. It is interesting that these ionic liquids are more hydrophilic than mineral oil, but this hydrophilicity in the mixture with graphite only permits the entry and leaving of the electrolyte but not a strong interaction with water molecules and between graphite particles. This is due to the strong non-covalent interactions between cation and anion, that avoid strong interactions with the surround. It is interesting to mention that the substituent effect of the cation in the electrochemical and impedance spectroscopical behavior is practically null.

## Figures and Tables

**Table 1:** ^1^H-chemical shifts (ppm) assignments for the ILs. (t: triplet, m: multiplet, d: doublet integration).

ILs	H-7	H-8	H-(9–13)	H-14	H-(17, 18)	H-(16, 19)	H-20
H-	4.60 (t, 2H)	1.99 (m, 2H)	1.33 (m, 10H)	0,86 (t, 3H)	8.79 (d, 2H)	8.06 (t, 2H)	8.47 (t, 1H)
CH_3_-	4.74 (t, 2H)	2.10 (m, 2H)	1.32 (m, 10H)	0.87 (t, 3H)	8.88 (d, 2H)	8.06 (d, 2H)	2.73 (t, 3H)
CN-	5.04 (t, 2H)	2.10 (m, 2H)	1.24 (m, 10H)	0.86 (t, 3H)	9.51 (d, 2H)	8.74 (d, 2H)	-
CF_3_-	4.76 (t, 2H)	2.04 (m, 2H)	1.27 (m, 10H)	0.89 (t, 3H)	9.12 (t, 3H)	8.27 (d, 2H)	-

**Table 2:** ^13^C-chemical shifts (ppm) assignments for the ILs.

ILs	C-7	C-8	C-9	C-(10, 11)	C-12	C-13	C-14	C-(17, 18)	C-(16, 19)	C-15	C-20
H-	62.46	30.67	25.41	28.19	32.42	22.24	14.20	145.44	128.65	144.37	-
CH_3_-	61.11	30.68	26.06	29.95	31.66	22.22	13.82	159.05	128.93	143.29	21.57
CN-	62.91	31.37	26.09	28.88	32.05	22.25	14.21	147.16	130.75	113.98	127.91
CF_3_-	63.51	31.49	25.84	28.77	31.55	22.44	13.87	145.92	125.18	118.10	122.03

**Table 3:** FT-IR frequency values obtained for the ILs.

ILs	C_sp_^2^-H (cm^−1^)	C_sp_^3^-H (cm^−1^)	C = N (cm^−1^)	C = C (cm^−1^)	Anion (cm^−1^)
H-	3125–3050	2934–2860	1653	1475	1353, 1045, 800, 610
CH_3_-	3122–3060	2930–2860	1656	1472	1366, 1042, 800, 608
CN-	3123–3055	2935–2864	1654	1478	1356, 1032, 805, 609
CF_3_-	3128–3051	2939–2861	1659	1471	1360, 1032, 805, 609

**Table 4:** Electroactive areas and roughness factor for all the systems. Data obtained from Equation (1) varying scan rates from 5 to 500 mV/s at pH 3 in a BR solution containing K_4_ [Fe (CN)_6_]/K_3_ [Fe (CN)_6_].

Electrode	Electroactive Area (cm^2^)	Roughness Factor (R)
CPE	0.032	1.03
H-	0.069	2.23
CH3-	0.079	2.55
CN-	0.080	2.58
CF3-	0.076	2.45

**Table 5:** The resistance of migration (Rs), the capacitance of the double layer (T), *p*-value, and resistance of Warburg (W_o_-R) obtained by fitting the equivalent circuit.

Electrode	R_s_ (Ω)	T (μF)	P	W_o_-R (Ω)
CPE	390.8 ± 15.8	0.16 ± 0.03	0.9777 ± 0.003	122,000 ± 6000
H-	269.1 ± 2.3	15.2 ± 0.8	0.9966 ± 0.02	37,257 ± 207
CH_3_-	129.2 ± 1.5	24.4 ± 2.1	0.8776 ± 0.03	16,333 ± 118
CN-	246.2 ± 2.8	47.4 ± 12.1	0.9104 ± 0.03	15,956 ± 263
CF_3_-	332.6 ± 2.8	97.4 ± 9.1	0.8419 ± 0.02	14,382 ±184

**Table 6:**
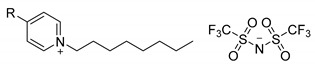
Relative Gibbs free energies (in kcal/mol) of the four different arrangements studied for each ionic liquid. Calculated at the PB86/def2-TZVP level, including D3BJ correction.

R	A	B	C	D
H-	1.23	0.04	3.02	0.00
CH_3_-	1.75	0.00	3.31	0.06
CN-	2.18	0.00	2.19	0.00
CF_3_-	2.48	0.83	1.61	0.00

**Table 7:** The number of non-covalent BCPs in the ionic liquids studied.

R	A	B	C	D
H-	9	11	7	9
CH_3_-	7	14	6	8
CN-	9	14	7	8
CF_3_-	9	15	8	8

**Table 8:** Averaged interaction energies (kcal/mol) for the ionic liquids studied and experimental melting points (°C).

R	E_int_	m.p.
H-	−79.39	−12
CH_3_-	−77.80	7
CN-	−83.77	28
CF_3_-	−82.04	30

## Supplementary Materials

The following are available online at https://www.mdpi.com/1420-3049/24/18/3382/s1. Figure S1: SEM micrographs of CPE and ILs. Figure S2: Water droplet assay.
