# Supplementary material for: Electrical and Electrochemical Behavior of Carbon Paste Electrodes Modified with Ionic Liquids Based in N-Octylpyridinium Bis(Trifluoromethylsulfonyl)Imide. A Theoretical and Experimental Study
Source: Molecules. 2019 Sep 17;24(18):3382. doi: 10.3390/molecules24183382 (PMC6767309; doi:10.3390/molecules24183382)
Supplement: Supplementary file 1 [file molecules-24-03382-s001.zip › Supplementary/Supplementary Materials.docx]

**Supplementary Materials**


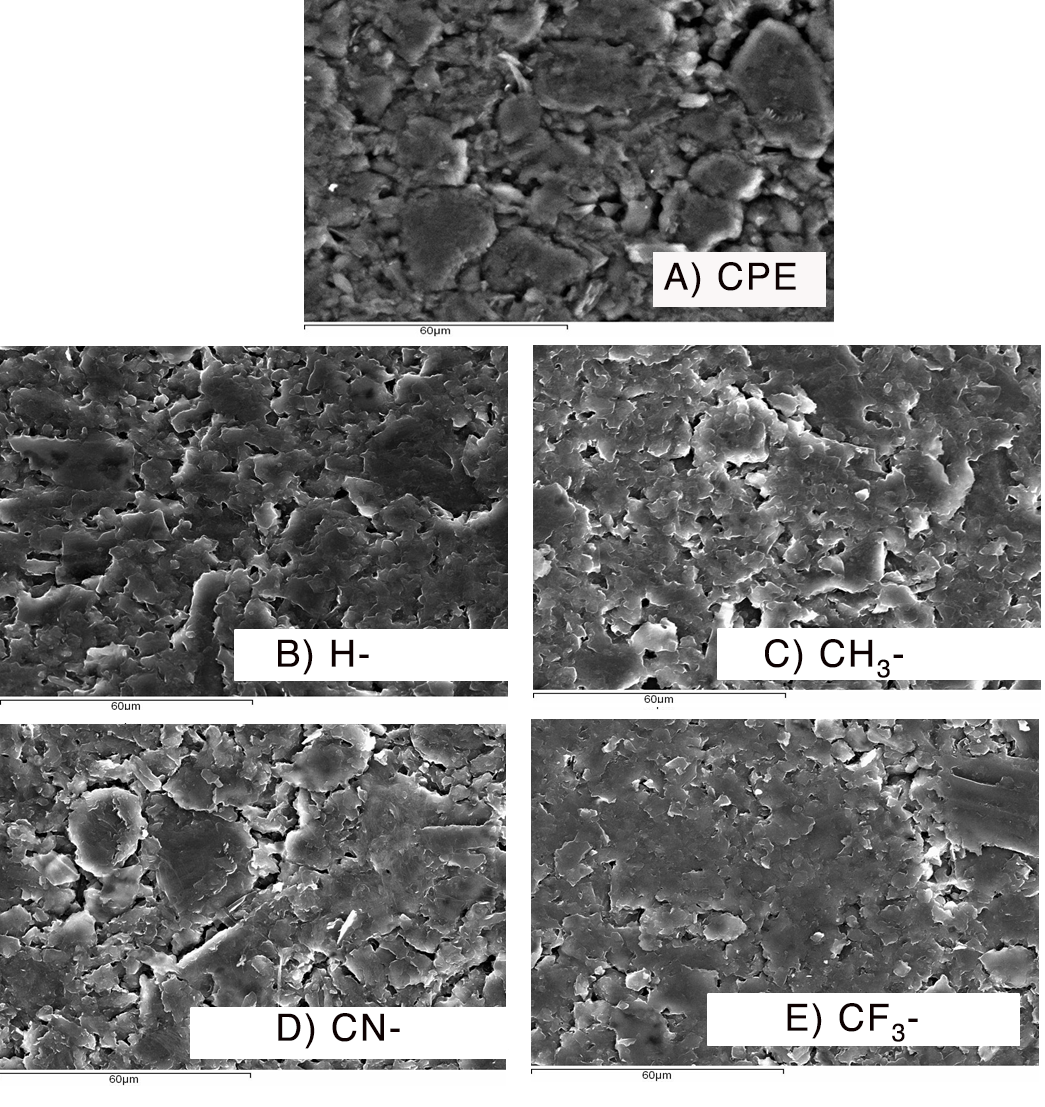


**Figure S1.** SEM micrographs. A) CPE and B, C, D, E are ILs-modified electrodes.


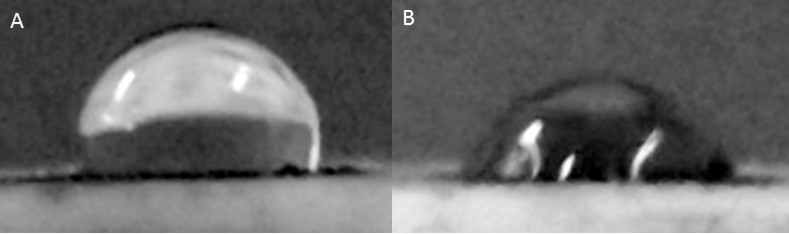


**Figure S2.** Water droplet assay. (**A**) CPE; (**B**) H-modified electrode. 3 µL water above the electrode surface was added.
