# Supplementary figures and images for: Electrical and Electrochemical Behavior of Carbon Paste Electrodes Modified with Ionic Liquids Based in N-Octylpyridinium Bis(Trifluoromethylsulfonyl)Imide. A Theoretical and Experimental Study
Source: Molecules. 2019 Sep 17;24(18):3382. doi: 10.3390/molecules24183382 (PMC6767309; doi:10.3390/molecules24183382)

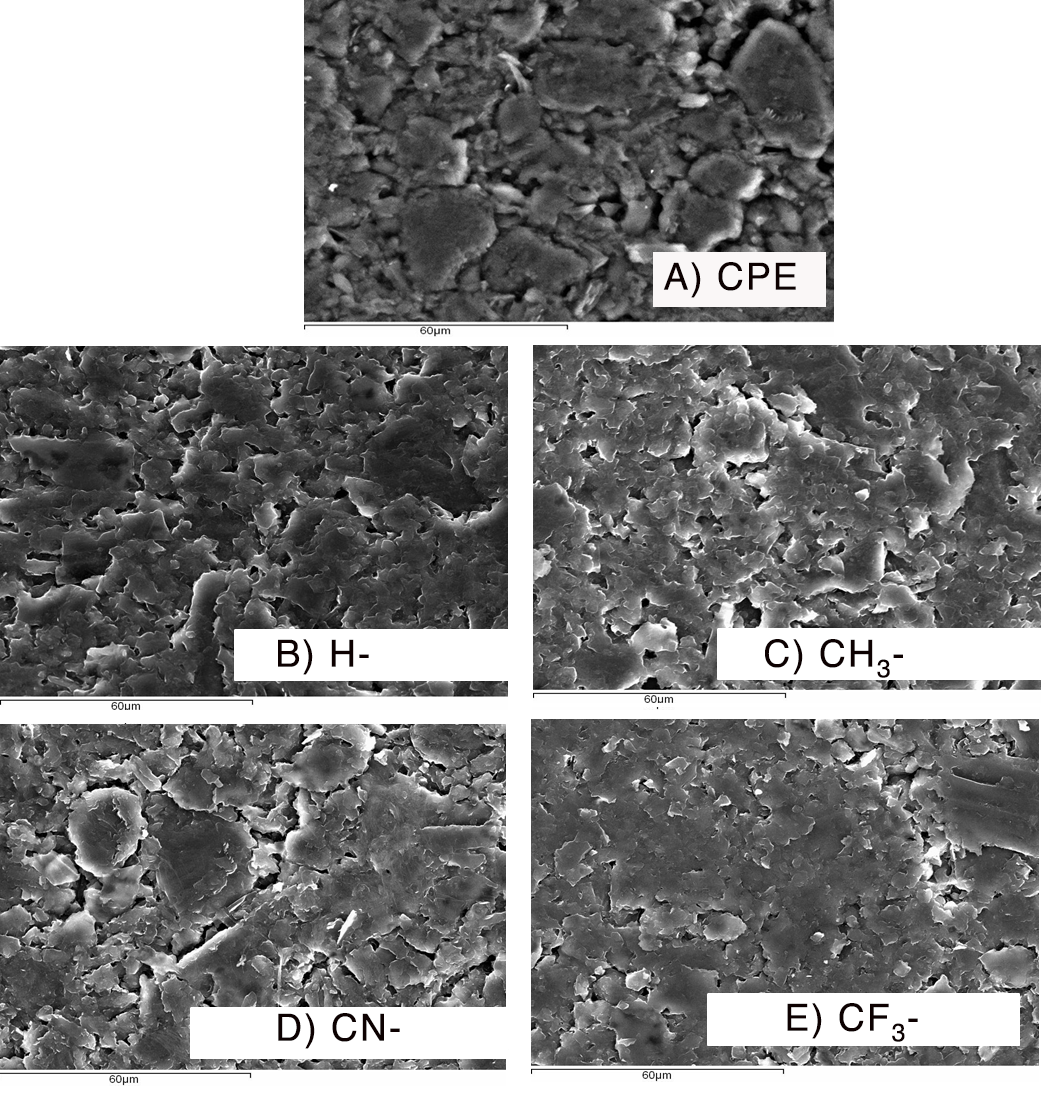

Supplement: Supplementary file 1 [file molecules-24-03382-s001.zip › Supplementary/Figure S1.png]

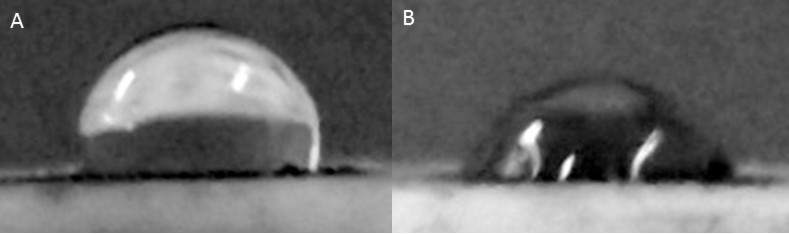

Supplement: Supplementary file 1 [file molecules-24-03382-s001.zip › Supplementary/Figure S2.jpg]
